# Does physical activity mediate the associations between blue space and mental health? A cross-sectional study in Australia

**DOI:** 10.1186/s12889-023-15101-3

**Published:** 2023-01-30

**Authors:** Emma Murrin, Nicole Taylor, Louisa Peralta, Dean Dudley, Wayne Cotton, Rhiannon Lee White

**Affiliations:** 1 School of Psychology, Western Sydney University, Locked Bag 1797, 2751 Penrith, NSW Australia; 2 School of Health Sciences, Western Sydney University, Locked Bag 1797, 2751 Penrith, NSW Australia; 3 Sydney School of Education and Social Work, The University of Sydney, 2006 Sydney, NSW Australia; 4 Macquarie School of Education, Macquarie University, 2109 Macquarie Park, NSW Australia

**Keywords:** Psychological well-being, Well being, Water, Exercise, Nature, Mediation

## Abstract

**Background:**

Research has begun to examine whether blue space is beneficial to mental health. While results are promising, it is difficult to know which aspects of mental health or mental ill-health may benefit most. Physical activity has been proposed as one potential mechanism via which blue space may be associated with better mental health. However, very few studies have examined mechanisms. We examined associations between blue space proximity and a range of mental health outcomes and examined which of these associations were mediated by physical activity.

**Methods:**

350 participants (*M* = 38.74, *SD* = 14.92, 70% female) self-reported their weekly physical activity and completed measures of depression, anxiety, and psychological wellbeing. We then used GIS software to calculate blue space proximity (i.e., coastal and inland), and structural equation modelling with mediation paths to determine the role of physical activity in the associations between bluespace and mental health.

**Results:**

Physical activity partially mediated the associations between coastal proximity and depression (β = 0.02, 95% CI = 0.001, 0.05), anxiety (β = 0.03, 95% CI = 0.01, 0.06), and wellbeing (β = − 0.03, 95% CI = − 0.08, − 0.01), and fully mediated the associations between inland water proximity and depression (β = 0.02, 95% CI = 0.003, 0.05), anxiety (β = 0.03, 95% CI = 0.01, 0.07), and wellbeing (β = − 0.03, 95% CI = − 0.07, − 0.01).

**Conclusion:**

While physical activity appears to explain associations between inland blue space and mental health outcomes, it only partially explains the association between coastal blue space and mental health, suggesting other mechanisms may play a role and even inactive exposure may be beneficial.

## Introduction

Mental health is a growing concern across all parts of the world with estimates of up to 400 million people living with some form of mental health issue [1]. Abundant research shows the value of nature (e.g., green space) to mental health [2], with more recent studies examining the potential benefit of blue space (i.e., natural or manmade outdoor environments that predominantly feature water; [3]). In one study of 16,307 participants spanning 18 countries, residential exposure to blue space was associated with wellbeing, and recreational visits to blue space (including inland and coastal waters) were associated with wellbeing, distress, anxiety, and depression [4].

A systematic review examining associations between green and blue spaces and mental health included 28 studies, yet only three observational studies examined blue space and mental health [5]. This highlights that evidence regarding blue space and mental health is lacking in comparison to evidence on green space and mental health. Nevertheless, of these studies, one study examined poor mental health, and risk of depression or anxiety, and identified no associations [6]. While this finding is important, this study only examined aspects of mental ill-health despite mental health and wellbeing being equally important to human health [7]. The other two studies both measured mental health via the General Health Questionnaire which includes symptoms of common mental health problems such as depression and anxiety [8]. Of these two studies one identified a significant association between blue space and mental health [9] and one did not [10]. While these studies were conducted in different geographic locations (i.e., England and Netherlands) with different access to blue space, the results suggest that findings are inconsistent. As such, further research is needed to better understand how blue space is associated with different psychological outcomes.

While research demonstrating the benefits of blue space for mental health is rapidly increasing, less research has sought to examine why blue space is beneficial to psychological outcomes. Nevertheless, three mechanisms have been proposed, including physical activity, social interaction, and mental restoration [11]. Given abundant evidence shows that physical activity is positively associated with mental health and is a key protective factor against depression and anxiety [12–15], it is certainly feasible that physical activity could be responsible for mental health benefits associated with blue space. Further, evidence shows that living closer to blue space is associated with significantly higher physical activity levels [16]. More specifically, living < 5 km from the coast in England has been associated with significantly higher odds of meeting physical activity guidelines [17]. However, research examining average distances between residence and the coast, across multiple countries, identified Australia an outlier in that individuals may live further from the coastline [18]. As such, the association between blue space proximity and physical activity may be different among Australians when compared to those living in the UK.

Interestingly, one study identified that those living closer to the coast reported better mental health, which was in part because they engaged in more outdoor physical activity such as walking [19]. While physical activity was a significant mediator of the association between coastal proximity and mental health, freshwater presence was directly associated with better mental health and this association was not mediated by physical activity [19]. This finding suggests that physical activity may play different mediating roles in different blue space contexts (e.g., coast or inland). Further, mental health was assessed via the General Health Questionnaire. While continually utilising this questionnaire to measure mental health facilitates comparability between studies [5], mental health is a broad construct that includes wellbeing, health, and a range of mental health disorders with varied symptoms, meaning blue space may hold different associations with different aspects of mental health. It is also possible there are different mechanisms for different psychological outcomes. For example, the mechanisms responsible for a reduction in depressive symptoms may be different to the mechanism responsible for promoting wellbeing. While several studies have examined emotional aspects of mental wellbeing such as happiness, and systematic review evidence of these studies has identified positive associations between greater exposure to outdoor blue spaces and mental wellbeing (N = 12 studies) [20], studies have not examined physical activity as a mediator of the association between blue space and wellbeing. Given anxiety disorders and mood disorders (e.g., depression) are the most common mental health disorders [21] and mental wellbeing is not only important for health but is also a protective factor against the onset of mental health disorders [7], we aimed to examine whether physical activity mediated associations between blue space proximity (i.e., coastal proximity and inland proximity) and depression, anxiety, and psychological wellbeing.

To achieve this aim, we developed two research questions: (1) is blue space proximity associated with mental health variables, and (2) does physical activity mediate any of these associations. Given there is copious literature showing inverse associations between green space and mental health disorders, and limited literature demonstrating similar associations between blue space and mental health disorders, it was hypothesised that proximity to blue space would be associated with lower symptoms of depression, anxiety, and psychological distress. Far less studies have examined the association between blue space and wellbeing, however limited research has shown that blue space is associated with positive emotions [22] and life satisfaction [23], therefore it was hypothesised that proximity to blue space would be associated with higher self-reported psychological wellbeing. Limited research has previously shown that physical activity mediated associations between blue space and poor mental health relationship, when measured as one unidimensional construct. In addition, abundant research has shown that physical activity is associated with depression, anxiety, and positive psychological wellbeing. Therefore, it was hypothesised that physical activity would mediate the association between blue space and both mental ill-health outcomes, and psychological wellbeing outcomes.

## Methods

### Study design

To begin to understand associations between blue space (predictor variable) and a broad variety of mental health outcome variables (i.e., depression, anxiety, and psychological wellbeing), we conducted an observational study. To understand the potential role that physical activity might play in these cross-sectional associations, we utilised structural equation modelling techniques to test for mediation.

### Participants

Based on a correlation coefficient of 0.15 to reflect the path from blue space to physical activity (*x* → *m*), a correlation of 0.18 to reflect the path from blue space to mental health (*x* → *y*), measured via the General Health Questionnaire, and a correlation of 0.37 for the path between physical activity and mental health (*m* → *y*) [24], to detect indirect effects with 0.80 power, a sample of 344 was required. To achieve a sample of this size, the questionnaire was published online through Qualtrics survey software, and anyone over the age of 18 years, who lived in NSW, Australia, was eligible to participate. The first question gained participants’ informed consent, and the questionnaire took an average of 16.76 min to complete. The study was approved by Western Sydney University Human Research Ethics Committee (H13804).

### Measures

#### Proximity to Blue space

To measure blue space proximity, we calculated the distance from each participant’s residential address to the nearest body of water. A body of water was defined in line with Australian Bureau of Statistics [25] Mesh Blocks coded as water. A Mesh Block is the smallest geographic unit of land defined by the Australian Bureau of Statistics [25] and can be classified as water, parkland, residential, industrial, commercial, education, hospital/medical, agricultural, or transport. A water Mesh Block indicates inland water (i.e., an aquatic environment located within land boundaries) and most commonly refers to a river, lake, or canal [26]. In line with previous research using Mesh Blocks to examine associations between blue and green spaces and health [27, 28], we then used Geographic Information Systems (GIS) software to calculate the shortest distance between two points. The first point being participants’ addresses and the second point being either: (a) the nearest water Mesh Block (i.e., inland blue space); or (b) the coast (i.e., the part of the land directly adjacent to the sea) [26]. Whichever of the two distances was shorter (i.e., coast proximity or proximity to the nearest inland water Mesh Block) represented proximity to the nearest blue space, however, we also retained the two separate variables (i.e., coastal proximity and inland water proximity) to examine associations between different types of blue space and mental health.

#### Depression

We used the Centre for Epidemiological Studies Depression Scale (CES-D-8) to measure symptoms of depression during the past week [29]. The scale consisted of the question “how often have you felt this way during the past week?” which was followed by 8 items reflecting symptoms of depression. Participants responded on a 4-point Likert scale ranging from 0 – *rarely or none of the time*, to 3 – *most or all of the time*. After recoding reverse scored items, total scores were calculated by summing all items, and ranged from 0 to 24, with higher scores reflecting higher rates of depressive symptoms. The CES-D-8 has strong psychometric properties among men and women, similar to the CESD-20 [29]. In the present study, internal consistency of this measure was *a* = 0.81.

#### Anxiety

We used the GAD-7 to measure generalised anxiety during the previous 2-weeks [30]. Participants responded to 7 items via a 4-point Likert scale from 0 – *not at all*, to 3 – *nearly every day*. Total scores ranged from 0 to 21 with 0–4 suggesting minimal generalised anxiety complaints and scores between 15 and 21 suggesting severe generalised anxiety. Previous studies demonstrate acceptable test-retest reliability (intraclass correlation = 0.83) [30]. In the present study, internal consistency of this measure was *a* = 0.91.

#### Wellbeing

To measure wellbeing, we used the PERMA-Profiler, which measures wellbeing in line with Seligman’s PERMA model [31]. This model includes five domains of wellbeing: positive emotion, engagement, relationships, meaning, and accomplishment, with three items used to assess each PERMA domain, and one item assessing happiness. Participants responded to the 16 items via an 11-point Likert scale from 0 (e.g., *never*) to 10 (e.g., *always*). Acceptable reliability has been shown for each of the domain subscales, and the total wellbeing score [32, 33]. In the present study, internal consistency values ranged from *a* = 0.74 to *a* = 0.92 for each subscale and internal consistency of the total wellbeing score was *a* = 0.94.

#### Physical Activity

Participants self-reported physical activity by completing the International Physical Activity Questionnaire - Short Form (IPAQ-SF). The IPAQ-SF asks participants to self-report the number of days (i.e., frequency) and minutes (i.e., duration) they engaged in walking, moderate activity, and vigorous activity, over the last 7 days [34]. After cleaning data, any reported minutes over 180 min were truncated at 180 min in line with reported maximum valid responses for each intensity. We then calculated MET-minutes/week by multiplying minutes and days by the corresponding intensity value [34]. We then summed the three intensity values to create a total MET-mins/week variable. The IPAQ-SF has been shown to have acceptable one-week test-retest reliability and moderate criterion validity using accelerometery as the standard [35].

### Data analysis

**Fig. 1:**
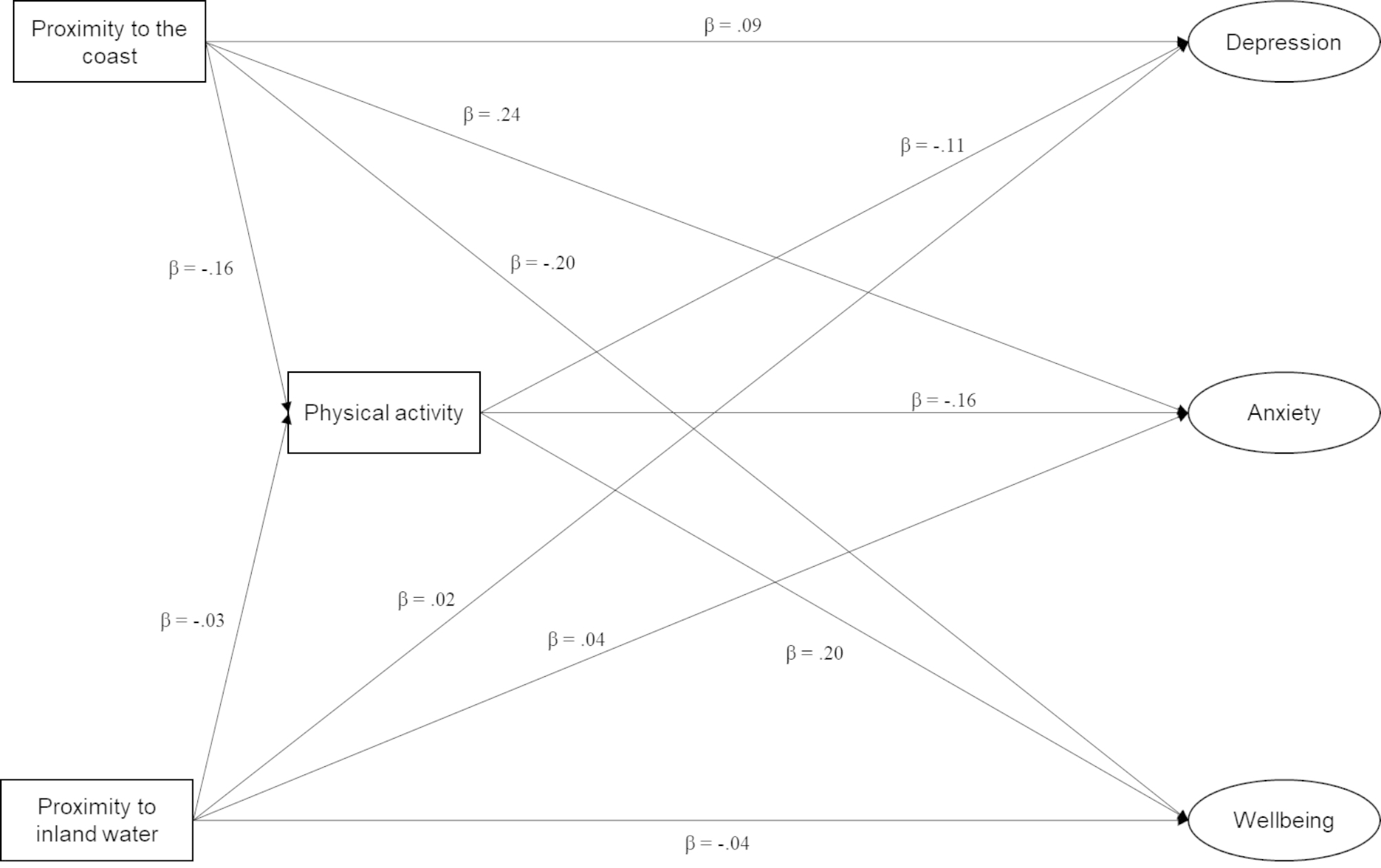
Standardised model estimates for full mediation structural equation model. (*p* values are not shown for readability, but all paths are statistically significant at *p* = < 0.05 as indicated by unbroken lines. Adjusted for age, sex, education, and income)

First, we calculated central tendency (means and standard deviations) for each variable. We then conducted a correlation matrix to determine underlying relationships between all variables. Because evidence shows that most distributions of physical activity data are positively skewed and not normally distributed [36], we examined the normality of the data before completing any analyses. Because variables were not normally distributed, we log transformed the data and conducted all analyses on the transformed data. To address research question 1, we first regressed all mental health variables on blue space proximity to identify associations between blue space and, depression, anxiety, and psychological wellbeing. We then repeated this step but with coastal proximity and inland water proximity separately to determine how different types of blue space were associated with different aspects of mental health. To examine research question 2, we then used structural equation modelling to conduct a mediation analysis with physical activity as the mediating variable (*m*), to determine indirect paths from both coast proximity and inland proximity (*x*) to mental health and wellbeing outcomes (*y*), through physical activity. In line with recommendations by Zhao [37], a significant indirect effect (i.e., the bootstrapped confidence intervals do not cross zero) suggests mediation. Also in line with Zhao [37], we reported the indirect effect and direct effect to determine the type of mediation, as well as the regression coefficients for each path to allow substantive interpretation of the results (see Fig. 1). Mediation analyses were conducted in Mplus [38] with 5,000 bootstrap iterations. Rather than replacing or inputting missing values, we used full information maximum likelihood estimation within the analysis to estimate a likelihood function for each participant based on available data so that all the available data are used in the model. All models also included age, sex, education status, and income as predictors in the SEM to account for possible confounding effects.

## Results

A total of 392 adults completed the online survey. Participants who did not report their address accurately (*N* = 42) were excluded, as this was required to calculate proximity to blue space. The remaining 350 participants (70% female) between 18 and 88 years old (*M* = 38.74, *SD* = 14.92) were included in all analyses. According to standard international cut offs that classify participants into three levels of physical activity [34], half (50%) of the participants reported engaging in high levels of physical activity per week, while 41.6% engaged in moderate levels of physical activity, and 8.4% engaged in low or no physical activity. Nearly half of the participants (48.6%) lived between one and five kilometres from blue space, while 20.1% lived between 5 and 10 km. Using the GAD-7 to measure anxiety, 63.1% of participants self-reported minimal anxiety while 3.8% reported severe anxiety. Half of the participants (52.9%) reported low levels of psychological distress, while 10.6% reported very high levels of distress. Table 1 reports the means and standard deviations of all variables.

**Table 1:** Correlations between all variables

	*M*	*SD*	Physical activity	Coast proximity	Inland Proximity	Depression	Anxiety
**Whole sample**							
Coast proximity	27.79	41.19	− 0.14*				
Inland Proximity	6.29	7.43	− 0.12*	0.48***			
Depression	7.79	4.66	− 0.07	0.08	− 0.02		
Anxiety	4.96	4.65	− 0.16**	0.15**	− 0.02	0.53***	
Wellbeing	7.71	1.59	0.20***	− 0.23***	− 0.05	− 0.46***	− 0.50***
**Males**							
Coast proximity	16.79	38.79	0.13				
Inland Proximity	5.80	3.54	− 0.04	0.20			
Depression	8.05	5.40	0.00	0.00	− 0.12		
Anxiety	3.54	3.59	− 0.08	0.04	− 0.13	0.38***	
Wellbeing	8.07	1.62	0.24*	− 0.39***	− 0.03	− 0.26**	− 0.41***
**Females**							
Coast proximity	30.04	37.04	− 0.14*				
Inland Proximity	6.03	6.95	− 0.15*	0.41***			
Depression	8.18	4.87	− 0.14*	0.12	0.02		
Anxiety	5.50	4.88	− 0.12	0.07	0.02	0.60***	
Wellbeing	7.56	1.55	0.13	− 0.14*	− 0.05	− 0.56***	− 0.51***

*p < .05, **p < .01, ***p < .001

### Blue Space and mental health

Proximity to blue space was significantly associated with anxiety (β = 0.18, 95% CI = 0.07, 0.37, *p* = .018) and wellbeing (β = − 0.14, 95% CI = − 0.26, − 0.02, *p* = .021), but not depression (β = 0.05, 95% CI = − 0.04, 0.14, *p* = .272). However, when examining relationships between mental health outcomes and the coast and inland water separately, proximity to the coast was significantly associated with depression (β = 0.18, 95% CI = 0.08, 0.32, *p* = .003), anxiety (β = 0.42, 95% CI = 0.25, 0.60, *p* = < 0.001), and wellbeing (β = − 0.27, 95% CI = − 0.48, − 0.09, *p* = .007). However, proximity to inland water was not significantly associated with depression (β = − 0.0918, 95% CI = − 0.21, 0.02, *p* = .110), anxiety (β = − 0.14, 95% CI = − 0.32, 0.02, *p* = .104), or wellbeing (β = 0.07, 95% CI = − 0.07, 0.20, *p* = .342).

### Indirect effects through physical activity

Results in Table 2 demonstrated a significant indirect effect between coastal proximity and depression (β = 0.02, 95% CI = 0.001, 0.05), anxiety (β = 0.03, 95% CI = 0.01, 0.06), and wellbeing (β = − 0.03, 95% CI = − 0.08, − 0.01). The direct effects for depression, anxiety, and wellbeing were also significant, suggesting complementary mediation where physical activity explains some of the association between coastal proximity and mental health.

Results also demonstrated significant indirect effects between inland water proximity and depression (β = 0.02, 95% CI = 0.003, 0.05), anxiety (β = 0.03, 95% CI = 0.01, 0.07), and wellbeing (β = − 0.03, 95% CI = − 0.07, − 0.01). However, the direct effects for depression, anxiety, and wellbeing were all non-significant, suggesting that inland proximity is only associated with mental health via physical activity.

**Table 2:** Mediation Structural Equation Model: Indirect effects through physical activity

Predictor	Outcome	Total effects				Direct effects				Indirect effects			
		β	SE	95% CI	*p*	β	SE	95% CI	*p*	β	SE	95% CI	*p*
Coast	Depression	0.13	0.04	0.05, 0.22	0.002	0.12	0.04	0.04, 0.20	0.006	0.02	0.01	0.001, 0.05	0.127
Coast	Anxiety	0.32	0.08	0.18, 0.49	0.000	0.30	0.08	0.15, 0.48	0.000	0.03	0.01	0.01, 0.06	0.063
Coast	Wellbeing	− 0.26	0.09	− 0.43, − 0.09	0.003	− 0.23	0.10	− 0.41, − 0.05	0.017	− 0.03	0.02	− 0.08, − 0.01	0.066
Inland	Depression	− 0.01	0.05	− 0.10, 0.09	0.910	− 0.02	0.05	− 0.12, 0.07	0.610	0.02	0.01	0.003, 0.05	0.084
Inland	Anxiety	0.02	0.08	− 0.17, 0.12	0.828	− 0.01	0.07	− 0.19, 0.10	0.850	0.03	0.01	0.01, 0.07	0.031
Inland	Wellbeing	− 0.03	0.08	− 0.15, 0.15	0.691	0.00	0.07	− 0.12, 0.17	0.964	− 0.03	0.02	− 0.07, − 0.01	0.034

Note: β = standardised coefficient, 95% CI = 95% bootstrapped confidence intervals with 5,000 iterations.

## Discussion

In addressing research question 1, the current study showed that living closer to blue space was generally associated with better wellbeing and lower symptoms of anxiety, but not lower symptoms of depression. While this result extends the findings of Gascon, Sánchez-Benavides [39] to an Australian population showing that blue space is not associated with depression, it contradicts findings by Dempsey, Devine [40] which showed a significant association between blue space and depression. It is important to note though that Dempsey, Devine [40] conceptualised blue space as visibility of coastal blue spaces. In further addressing associations between blue space and mental health, this study showed that living near the coast was associated with improved wellbeing, and lower anxiety and depression. However, these associations did not exist for those living near inland blue space. This suggests that different types of blue space locations (e.g., coast versus inland) hold different associations with mental health. Nevertheless, the findings of this study generally suggest that blue space may be a beneficial tool in mental health promotion (i.e., promoting positive emotion, meaning, accomplishment, and happiness) and protection (i.e., preventing or alleviating psychological distress and symptoms of anxiety). And indeed, research shows great acceptability for mental health services to integrate recommendations around utilising nature to improve mental health [41]. However, longitudinal studies are required to determine causation and examine whether exposure to blue space can significantly improve mental health, and if so, if one needs to view, be in, or be near water. Further, future studies could consider using wearable camera technology to capture and measure actual exposure to visible water rather than proximity. This would enable better understanding of how blue spaces may or may not be useful in treating or preventing depression and anxiety and promoting wellbeing.

In answering research question 2, results of the current study showed that physical activity mediated the associations between blue space and, depression, anxiety, and wellbeing (i.e., conceptualised as positive emotion, engagement, relationships, meaning, accomplishment, and happiness). This may be unsurprising given studies show that mood increases after exercise [42], and extensive evidence shows that leisure-time physical activity is positively associated with mental health and wellbeing [43]. There are also long-established associations between physical activity and; life satisfaction, meaning, optimism, and a sense of accomplishment [44]. These psychological benefits of physical activity appear to explain the association between blue space and wellbeing. However, physical activity only partially mediated the association between coastal blue space and mental health outcomes (i.e., complementary mediation). This means that other innate aspects of blue space itself, or another mechanism besides physical activity, must also enable coastal water environments to benefit mental health [37].

Rumination is a maladaptive self-focused emotion regulation strategy that involves persistent and repetitive analysing of reasons for negative moods, and is a key risk factor for the onset of mental illnesses [45, 46]. Bratman et al. [47] however, suggested that *awe* experiences facilitated by engagement with nature can improve mood. Further, research has shown that coastal blue space exposure resulted in increased feelings of calm, relaxed, and rejuvenated [22]. And qualitative findings also allude to feeling calm (e.g., *“Just head down to the water, it just seems to calm your mind”)* [48] and free (*e.g., “I’m myself, as free as a bird … I’m not angry with myself”)* [49]. Therefore, it is plausible that a sense of *awe* may be a mechanism via which coastal blue space is associated with better mental health, in addition to physical activity.

Interestingly though, there was no direct association between inland blue space and mental health, and indirect-only mediation. This means that physical activity levels are likely to explain why living nearer to inland water is associated with improved wellbeing and lower symptoms of depression and anxiety (i.e., indirect-only mediation; [37]). This suggests that people may need to be active when visiting inland blue space locations to experience mental health benefits, and perhaps awe experiences are not as common at inland locations when compared to the coast. Kaplan’s attention restoration theory explains that natural environments, more so than urban settings, promote psychological distance from one’s usual context and thereby promote attention restoration (i.e., psychological recovery from fatigue associated with directed mental effort) and foster a feeling of *being away* [50]. Coastal views may simply provide this sense of being away more than inland views because of the concept of the coastline. Nevertheless, these findings contradict previous mediation research which showed that that freshwater proximity was directly associated with mental health and not mediated by physical activity [19]. While this finding might contradict the current results, inland freshwater blue spaces vary drastically between countries, and between different geographic areas in the same country, and perhaps more specific classification is needed to better understand mechanisms in the future.

### Strengths and limitations

The most notable strength of this study was the inclusion of a range of mental health related constructs in one study (i.e., depression, anxiety, and psychological wellbeing). This enabled us to understand how blue space proximity (coastal and inland) is differently associated with different aspects of mental health and wellbeing. Testing whether physical activity mediated these relationships was also a strength of the current study, as no study has examined physical activity across these different relationships, despite it being suggested as a potential mechanism. Despite several strengths, there are a number of limitations. First, we only measured proximity to blue space. While those who live closer to blue space have increased access to blue space, this does not necessarily equate to higher use of, or exposure to, blue space. Additionally, we did not examine changes in mental health. Future research needs to measure proximity and mental health longitudinally and examine effects for those who move closer to, or further from blue space, to better understand the role proximity plays. Additionally, while coastal blue spaces may be more similar in nature, inland blue spaces can range from lakes, to rivers, to much smaller ponds and watering holes. While they all contain freshwater and are all bound by land, they may include a vast array of visual features that vary significantly from the coast, and potentially also from each other. Because we only measured proximity to inland blue space, we cannot distinguish relationships between different types of inland blue space. Future research could consider measuring environmental and visual aspects of different blue spaces to determine which aspects are associated with greater mental health benefits. Finally, while physical activity mediated the associations between blue space proximity and mental health, longitudinal studies are required to determine cause and effect. It is also imperative that future studies examine a range of potential mediators, including auditory, visual, social, and psychological mechanisms, particularly in relation to coastal blue spaces. These studies would enable us to better recommend the specific activities or behaviours that are most likely to be beneficial for different mental health. Finally, the sample is limited to participants in NSW, Australia, who were able to complete an online survey. Therefore, findings may not be generalisable to other geographic locations. Also, participants may be from higher socioeconomic backgrounds or younger demographics due to the nature of online data collection. Therefore, future research should specifically examine these associations among older people using more accessible methods of data collection.

### Practical applications and future research

Nature exposure has long been recommended to improve mental health [51] and has even been acknowledged as appropriate for high-risk populations [52]. Despite increasing research on blue space, a recent World Health Organization review of both green and blue space reported that research on blue space is limited in comparison, especially for inland waters [26]. This study however, showed that coastal blue space proximity was directly associated with better mental health, while inland blue space exposure was associated with mental health via increased physical activity. Given abundant research demonstrates leisure-time physical activity is associated with better mental health [43], leisure-time physical activity near inland blue space environments such as rivers could be promoted. However, further research on types of inland locations, auditory and visual factors that are essential, and social and psychological mechanisms need to be understood before more concrete recommendations can be made. Nevertheless, research shows that mental health practitioners are receptive to recommending park-based physical activity [53], and this could indeed be extended to blue space environments if further research is conducted.

## Conclusion

Interestingly, those who live closer to blue space report higher levels of wellbeing and lower levels of depression and anxiety. Further, physical activity mediated these associations. However, physical activity partially explained the associations between the coast and mental health, and fully mediated (indirect-only) the associations between inland blue space and mental health. As such, it is likely that psychological, environmental, and social mechanisms mediate the association between coastal proximity and mental health, in addition to physical activity. However, longitudinal research is needed to establish cause and effect relationships. Further, better understanding which mechanisms play a role in different contexts would enable us to better utilise blue space locations within mental health treatment and promotion. Therefore, future research should consider employing longitudinal studies that examine a broader variety of blue space types and multiple mediators, to better understand how psychological, environmental, and social mechanisms interact in different blue space contexts. Nevertheless, from the current results, we can conclude that those who live nearer to inland blue space in NSW Australia, are likely to be more active, and experience a greater sense of wellbeing and lower depression and anxiety as a result.

## Data Availability

The datasets generated and/or analysed during the current study are not publicly available due to ethical constraints but are available from the corresponding author on reasonable request.
